# Negative lymph node count is an independent prognostic factor for patients with rectal cancer who received preoperative radiotherapy

**DOI:** 10.1186/s12885-017-3222-8

**Published:** 2017-03-28

**Authors:** Xinxing Li, Hao Lu, Kai Xu, Haolu Wang, Xiaowen Liang, Zhiqian Hu

**Affiliations:** 1 Department of General Surgery, Changzheng Hospital, The Second Military Medical University, 415 S. Fengyang Road, Shanghai, 200003 China; 2 Therapeutics Research Centre, School of Medicine, The University of Queensland, Princess Alexandra Hospital, Woolloongabba, QLD 4102 Australia

**Keywords:** Rectal cancer, Preoperative radiotherapy, Negative lymph node, Survival

## Abstract

**Background:**

Negative lymph node (NLN) count has been reported to provide more accurate prognostic information than the N stage alone in patients with rectal cancer (RC). Since preoperative radiotherapy (Pre-RT) can significantly affect the LN status, it is unclear whether NLN count still has prognostic value count on survival of patients with RC who received Pre-RT.

**Methods:**

In this study, clinicopathological characteristics, number of positive LNs and survival time were collected from Surveillance, Epidemiology, and End Results Program (SEER)-registered RC patients. Univariate and multivariate Cox proportional hazards models were used to assess the risk factors for survival.

**Results:**

X-tile plots identified 9 (*P* < 0.001) as the optimal cutoff NLN value to divide the patients into high and low risk subsets in terms of cause specific survival (CSS). NLN count was validated as independently prognostic factor in univariate and multivariate analysis (*P* < 0.001). Subgroup analysis showed that NLN count was an independently prognostic factor for patients with stage ypII (*P* = 0.002) and ypIII (*P* < 0.001).

**Conclusions:**

Our results firmly demonstrated that NLN count provides accurate prognostic information for RC patients with Pre-RT.

## Background

Rectal cancer (RC) is one of the most common malignancies in the USA, and the incidence of RC in Asian countries is increasing rapidly and has been considered to be similar to that of the Western countries [1]. Preoperative radiotherapy (Pre-RT) has become part of standard practice offered to improve treatment outcomes in patients with RC because of the oncologic benefit of reduced local recurrence rate [2]. But till now, there is still lack of effective means for accurate prognostic evaluation on the survival.

It is widely thought that lymph node (LN) metastasis indicates worse tumor response grade and poorer survival. According to the guidelines for RC from the National Comprehensive Cancer Network, a minimum of twelve lymph nodes must be retrieved and examined for accurate staging and the number of metastatic LNs was validated as an independent prognostic factors [3]. While the node-positive patients with RC are heterogeneous and the prognosis of these patients cannot be stratified by the node-stage only [3, 4]. Therefore, the concept of negative lymph node (NLN) counts has attracted attention recently as a prognostic indicator in various cancers, including breast [5], cervical [6], and esophagus [7]. It has been reported that the number of NLNs was an independent prognostic factor for patients with colon cancer [8]. However, Pre-RT can yield tumor downstaging, reduce the burden of residual microscopic disease at surgery and reduce the number of LNs retrieved in operation [9]. With the decreased LNs retrieval, the prognostic value of the LN count might also diminish [10]. It has been reported that increased number of NLNs is associated with improved survival in pathological IIIB and IIIC RC treated with Pre-RT [7]. While it is still unclear whether NLN still has prognostic value count on survival of all patients with RC, including stage I and II, who received Pre-RT. The purpose of this study was to assess the association between NLN count and survival of patients with RC of all stages who received Pre-RT. In order to get convincing results in a larger series patients, we used the SEER (Surveillance, Epidemiology and End Results)-registered database to analyze this association, and determine the optimal cutoff value of NLN count.

## Methods

### Study population and data extracted

The SEER (Surveillance, Epidemiology, and End Results Program) database and SEER-stat software (SEER∗Stat 8.3.2) were used to identify patients whose pathological diagnosis as RC between 2004 and 2010. Only patients who underwent Pre-RT and surgical treatment with age of diagnosis more than 18 years were included. Histological type were limited to adenocarcinoma (8140/3), carcinoma (8010/3; 8020/3; 8021/3 and 8145/3) and signet ring cell carcinoma (8490/3). Patients were excluded if they had only local excision following Pre-RT, multiple primary malignant neoplasms, incomplete TNM staging, with distant metastasis (M1), no evaluation on LNs, died within 30 days after surgery or information on CSS and survival months unavailable.

Year of diagnosis, age, sex, race, grade, histologic type, ypT stage, number of LNs examined, number of positive LNs and CSS were assessed. TNM classification was restaged according to the criteria described in the AJCC Cancer Staging Manual (7th edition, 2010).

### Statistical analysis

The NLNs cutoff points were determined using the X-tile program, which identified the cutoff value with the minimum *P* values from log-rank χ2 statistics for the categorical NLNs in terms of CSS. Baseline characteristics were compared using the X2 test for nominal variables. Survival curves were generated using Kaplan-Meier analyses, and the differences between the curves were analyzed by log-rank test. Cox regression models were built for analysis of risk factors for survival outcomes. Statistical analyses were performed using the statistical software package SPSS for Windows, version 19.0 (SPSS Inc., Chicago, IL). All *P* values were two-sided. *P* < 0.05 was considered statistically significant.

## Results

### Patient characteristics from SEER database

In SEER database, we selected a total of 6068 patients with RC who received Pre-RT, including 3881 male and 2187 female from 2004 to 2010. The median age of patients was 59 years. There were 4042 patients with stage ypN0, 1352 with stage ypN1 and 674 with stage ypN2.

### The optimal cutoff value for NLN count calculated by X-tile

**Fig. 1:**
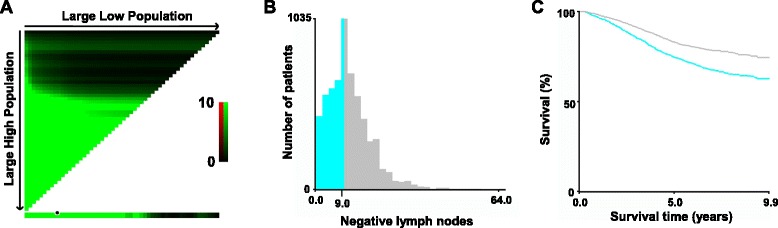
X-tile analysis of survival data from the SEER registry. X-tile analysis was performed using patient data, equally divided into training and validation sets, from the SEER registry. In X-tile plots of the training sets (**a**), the plots of matched validation sets are shown in the smaller inset. The optimal cut-point highlighted by the *black circle* (**a**) is shown on a histogram of the entire cohort (**b**), and a Kaplan-Meier plot (**c**). *P* values were determined using the cutoff point defined in the training set and applying it to the validation set. (The optimal cutoff value for NLN count is 9, χ2 = 78.060, *P* < 0.001)

To assess the influence of different NLN count on CSS, we analyzed the individual result using different NLN count ranging from 0 to 23. The 5-year CSS was calculated for patients with N (NLNs number) or more nodes and less than N nodes. As shown in Table 1, NLN count in patients with RC who received Pre-RT was a prognosis factor for number ranging from 0 to 23. With the NLN count increased from 0 to 23, the 5-year CSS rate increased from 51.8 to 88.8% (Table 1). Next X-tile plots were constructed and the maximum χ2 log-rank value of 78.060 (the number as 9, Fig. 1, *P* < 0.001) was produced, applying 9 as the optimal cutoff value to divide the cohort into high and low risk subsets in terms of CSS. There was a significant improvement in 5-year CSS between two subsets (74.3% v.s. 82.8%, Table 1). As shown in Table 2, the year of diagnosis, sex, age and the average number of LNs dissected were significantly different between patients with NLN ≤ 9 or >9 (*P* < 0.05).

**Table 1:** Univariate analysis of the influence of different NLN count on CSS in RC patients who received Pre-RT

LNs	No.	5-year CCS	χ2	*P* value	LNs	No.	5-year CCS	χ2	*P* value
≤0	72	51.8%	41.745	0.000	≤12	3685	76.5%	42.763	0.000
>0	5996	79.5%			>12	2383	83.3%		
≤1	230	63.5%	50.100	0.000	≤13	4022	76.9%	38.747	0.000
>1	5838	79.7%			>13	2046	83.6%		
≤2	444	68.2%	44.132	0.000	≤14	4320	77.3%	33.939	0.000
>2	5624	80.0%			>14	1748	83.7%		
≤3	720	69.0%	54.496	0.000	≤15	4585	77.3%	36.435	0.000
>3	5348	80.5%			>15	1483	84.8%		
≤4	1015	70.0%	67.375	0.000	≤16	4822	77.6%	33.037	0.000
>4	5053	81.0%			>16	1246	85.3%		
≤5	1314	71.8%	68.432	0.000	≤17	5013	72.8%	27.827	0.000
>5	4754	81.2%			>17	1055	85.4%		
≤6	1625	72.5%	74.979	0.000	≤18	5177	78.0%	23.546	0.000
>6	4443	81.6%			>18	891	85.5%		
≤7	1974	73.5%	74.302	0.000	≤19	5302	78.0%	25.448	0.000
>7	4094	81.9%			>19	766	86.8%		
≤8	2285	74.2%	70.405	0.000	≤20	5422	78.2%	25.257	0.000
>8	3783	82.1%			>20	646	87.2%		
≤9	2613	74.3%	78.060	0.000	≤21	5528	77.9%	21.120	0.000
>9	3455	82.8%			>21	540	87.5%		
≤10	2973	75.0%	67.236	0.000	≤22	5606	78.4%	21.730	0.000
>10	3095	83.1%			>22	462	88.0%		
≤11	3320	75.6%	57.663	0.000	≤23	5671	78.5%	18.872	0.000
>11	2748	83.4%			>23	397	88.8%

**Table 2:** Comparison of clinical characteristics of patients with NLN ≤ 9 or NLN > 9

Characteristic		Total	NLN ≤ 9	NLN > 9	*P* value
		6068	2613	3455	
Follow up	Median (IQU)	58 (42-81) months			
Year of diagnosis					0.000
	2004–2007	3122	1553	1569	
	2008–2010	2946	1060	1886	
Sex					0.007
	Male	3881	1625	2256	
	Female	2187	988	1199	
Age					0.000
	<60	3368	1365	2003	
	≥60	2700	1248	1452	
Race					0.593
	White	4927	2137	2790	
	Black	511	214	297	
	Others	630	262	368	
Grade					0.222
	Grade I/ II	5098	2184	2914	
	Grade III/ IV	970	429	541	
Histologic type					0.430
	Adenocarcinoma	5517	2367	3150	
	Mucinous adenocarcinoma	490	215	275	
	Signet ring cell carcinoma	61	31	30	
ypT stage		386	189	197	0.062
	1	957	425	532	
	2	4306	1820	2486	
	3	419	179	240	
	4				
LNs dissected		12.79	6.92	17.23	0.000

### Impact of the NLN count on CSS in RC patients who received Pre-RT

According to univariate analysis, age (≥ 60), race (black persons), grade (III/IV), histologic type (signet ring cell carcinoma), ypT stage (ypT3 and ypT4) and NLNs (≤ 9) were associated with poor outcomes in patients with RC who received Pre-RT (*P* < 0.001) (Table 3). In multivariate Cox analysis, age, grade, histologic type, ypT stage and NLN counts were independently prognostic factors (*P* < 0.001, Table 3). NLN counts (> 9) exhibited a favorable effect on survival (HR = 1.623, 95% CI 1.457 ~ 1.809, *P* < 0.001, Table 3).

**Table 3:** Univariate and multivariate survival analyses of RC patients who received Pre-RT

Characteristic	5-year CCS	Univariate analysis		Multivariate analysis	
		χ2 test	*P*	HR(95%CI)	*P*
Year of diagnosis		2.412	0.120	NI	
2004–2007	78.4%				
2008–2010	80.2%				
Sex		1.655	0.198	NI	
Male	29.5%				
Female	78.4%				
Age		21.805	0.000		0.000
< 60	81.3%			Ref	
≥ 60	76.4%			0.777 (0.697 ~ 0.866)	0.000
Race		14.873	0.000		0.909
White	79.5%			Ref	
Black	72.7%			1.129 (0.937 ~ 1.364)	0.202
Others	81.8%			1.585 (1.246 ~ 2.017)	0.000
Grade		112.363	0.000		0.000
Grade I/ II	81.5%			Ref	
Grade III/ IV	66.9%			0.596 (0.523 ~ 0.680)	0.000
Histologic type		81.128	0.000		0.000
Adenocarcinoma	80.4%			Ref	
Mucinous adenocarcinoma	69.2%			0.412 (0.308 ~ 0.635)	0.000
Signet ring cell carcinoma	32.5%			0.601 (0.408 ~ 0.885)	0.010
ypT stage		148.323	0.000		0.000
1	88.6%			Ref	
2	87.9%			0.293 (0.212 ~ 0.405)	0.000
3	77.9%			0.303 (0.239 ~ 0.384)	0.000
4	62.6%			0.584 (0.494 ~ 0.692)	0.000
LNs		2.234	0.135	NI	
≤ 12	78.5%				
> 12	79.8%				
NLN		78.060	0.000		0.000
≤ 9	74.3%			Ref	
> 9	82.8%			1.623 (1.457 ~ 1.809)	0.000

### Impact of the NLN count on CSS in RC patients based on each pathological grade

**Fig. 2:**
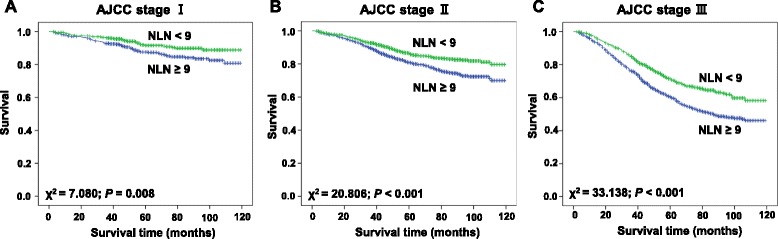
Log-rank tests of CSS comparing patients with NLNs (<9 VS ≥9) for (**a**) stage ypI: *P* = 0.008; (**b**) stage ypII: *P* < 0.001; and (**c**) stage ypIII: *P* <0.001

We then further analyzed of the effects of NLN on survival in each subgroup of stage yp I, yp II and yp III. As shown in Fig. 2, subgroup analysis showed that NLN was a prognosis factor for patients with RC who received Pre-RT in stage ypI (χ2 = 7.080; *P* = 0.008), ypII (χ2 = 20.806; *P* < 0.001) and ypIII (χ2 = 33.138; *P* < 0.001), respectively. Moreover, as shown in Tables 4, 5 and 6, the NLN count was also an independently prognostic factor in stage ypII (HR: 1.520, 95%CI: 1.170 ~ 1.975; *P* = 0.002) and ypIII (HR: 1.466, 95%CI: 1.264 ~ 1.700; *P* < 0.001) after adjustment for potential confounders.

**Table 4:** Univariate and multivariate survival analyses of stage ypI RC patients who received Pre-RT

Characteristic	5-year CCS	Univariate analysis		Multivariate analysis	
		χ2 test	*P*	HR(95%CI)	*P*
Year of diagnosis		6.324	0.012		0.035
2004–2007	87.9%			Ref	
2008–2010	92.2%			1.609 (1.029 ~ 2.517)	0.037
Sex		1.238	0.266	NI	
Male	90.0%				
Female	89.3%				
Age		2.359	0.125	NI	
< 60	91.3%				
≥ 60	88.0%				
Race		6.222	0.045		0.053
White	88.9%			Ref	
Black	81.0%			3.792 (1.201 ~ 11.968)	0.023
Others	98.9%			4.392 (1.251 ~ 15.415)	0.021
Grade		0.483	0.487	NI	
Grade I/ II	90.0%				
Grade III/ IV	85.1%				
Histologic type		3.218	0.200	NI	
Adenocarcinoma	89.8%				
Mucinous adenocarcinoma	89.5%				
Signet ring cell carcinoma	50.0%				
ypT stage		0.001	0.972	NI	
1	90.5%				
2	89.4%				
LNs		9.401	0.002		0.284
≤ 12	87.8%			Ref	
> 12	93.4%			1.702 (0.949 ~ 3.052)	0.074
NLN		7.080	0.008		0.600
≤ 9	87.6%			Ref	
> 9	91.6%			1.128 (0.682 ~ 1.866)	0.638

**Table 5:** Univariate and multivariate survival analyses of stage ypII RC patients who received Pre-RT

Characteristic	5-year CCS	Univariate analysis		Multivariate analysis	
		χ2 test	*P*	HR(95%CI)	*P*
Year of diagnosis		0.255	0.614	NI	
2004–2007	84.1%				
2008–2010	84.7%				
Sex		1.414	0.234	NI	
Male	85.1%				
Female	83.0%				
Age		21.435	0.000		0.000
< 60	87.2%			Ref	
≥ 60	81.1%			0.677 (0.566 ~ 0.810)	0.000
Race		1.812	0.404	NI	
White	84.6%				
Black	81.4%				
Others	84.4%				
Grade		24.552	0.000		0.000
Grade I/ II	85.6%			Ref	
Grade III/ IV	76.2%			0.599 (0.479 ~ 0.748)	0.000
Histologic type		6.705	0.035		0.042
Adenocarcinoma	85.0%			Ref	
Mucinous adenocarcinoma	75.9%			0.786 (0.249 ~ 2.481)	0.682
Signet ring cell carcinoma	71.6%			1.103 (0.339 ~ 3.590)	0.871
ypT stage		21.301	0.000		0.000
3	85.4%			Ref	
4	73.2%			0.591 (0.445 ~ 0.767)	0.000
LNs		9.648	0.002		0.892
≤ 12	82.5%			Ref	
> 12	86.7%			0.983 (0.749 ~ 1.289)	0.900
NLN		20.806	0.000		0.002
≤ 9	80.9%			Ref	
> 9	86.5%			1.520 (1.170 ~ 1.975)	0.002

**Table 6:** Univariate and multivariate survival analyses of stage ypIII RC patients who received Pre-RT

Characteristic	5-year CCS	Univariate analysis		Multivariate analysis	
		χ2 test	*P*	HR(95%CI)	*P*
Year of diagnosis		0.038	0.846	NI	
2004–2007	65.6%				
2008–2010	67.0%				
Sex		0.026	0.872	NI	
Male	65.9%				
Female	66.2%				
Age		17.245	0.000		0.000
< 60	69.3%			Ref	
≥ 60	60.9%			0.747 (0.654 ~ 0.866)	0.000
Race		17.535	0.000		0.625
White	66.8%			Ref	
Black	51.6%			1.125 (0.880 ~ 1.436)	0.347
Others	71.3%			1.767 (1.290 ~ 2.421)	0.000
Grade		41.722	0.000		0.000
Grade I/ II	69.5%			Ref	
Grade III/ IV	53.9%			0.670 (0.566 ~ 0.794)	0.000
Histologic type		24.617	0.000		0.037
Adenocarcinoma	67.4%			Ref	
Mucinous adenocarcinoma	60.7%			0.562 (0.378 ~ 0.836)	0.004
Signet ring cell carcinoma	41.8%			0.588 (0.383 ~ 0.904)	0.016
ypT stage		66.241	0.000		0.000
1	73.0%			Ref	
2	83.7%			0.442 (0.242 ~ 0.810)	0.008
3	65.1%			0.276 (0.195 ~ 0.391)	0.000
4	46.8%			0.564 (0.451 ~ 0.704)	0.000
LNs		1.975	0.160	NI	
≤ 12	64.9%				
> 12	67.0%				
NLN		33.138	0.000		0.000
≤ 9	60.5%			Ref	
> 9	71.4%			1.466 (1.264 ~ 1.700)	0.000

## Discussion

LN metastasis is considered as one of the most significant prognostic factors in RC [11]. The total number of LNs retrieved is fundamental in most pathological staging systems for RC. Inadequate LN evaluation is associated with worse outcomes in terms of tumor recurrence and patient survival [12]. Thus, looking for effective means of assessment of LNs on survival can provide more accurate prognostic information.

According to the AJCC-7 RC staging system, a 12-node minimum is required for proper tumor stage and associated with a good survival outcome in patients treated with surgery [3]. Yet, the number of positive LN is often affected by many facts such as neoadjuvant therapy, and the number of LN retrieved and inspected [3]. Once the range of LN retrieved is not enough, the prediction of survival would be inaccurate. Therefore, the concept of NLN has been developed recently. NLN count has a unique advantage that it is little influenced by the number of LN retrieved [13]. The more NLN count is, the better the survival would be. Li et al. [12] reported that the optimal cutoff value of 10 was validated as an independent prognostic factor in stage ypIIIB and ypIIIC RC patients treated with Pre-RT. In this study, we found that the NLN count was an independent prognosis factor for all patients with RC who received Pre-RT. And we identified the optimal cutoff value for NLN count as 9. Obviously, NLN count is a good supplement for LN stage and TNM stage on evaluating the prognosis of patients with RC who received Pre-RT, respectively for patients with stage ypI, ypII and ypIII. Until now, there has been no report confirming the mechanism of NLNs influencing on the prognosis of RC. But it is suggested that lymphatic micrometastasis is a key etiology of recurrence and metastasis after resection of RC [14]. LN micrometastasis, is common in nodes with the size ranging from 0.2 to 2.0 mm which determined to be negative by HE staining, but positive for cytokeratin (CK) by immunohistochemical staining [15]. Because it is difficult to find lymphatic micrometastasis during operation, we can retrieve more NLNs to reduce the residual micrometastases, in order to improve the prognosis of RC.

The intended purpose of Pre-RT is tumor down-staging by decreasing the primary tumor bulk and reducing the burden of residual associated LN metastases at surgery, thus increasing the potential of margin-negative resections [9]. It has been reported that Pre-RT may cause radiation-induced lymphocyte destruction and stromal fibrosis resulting in alterations of the morphology of the LNs, making LN detection during operation more difficult [9]. Some researchers found that a decreased LN count after Pre-RT was related to good survival [16, 17]. The NLN count is particularly useful in the prediction of survival because it is little influenced by the number of LN retrieved, and has potential to reflect the dissection of lymphatic micrometastasis. In this study, we revealed that NLN count was an independent prognostic factor for patients with RC who received Pre-RT. Subgroup analysis showed that NLN was a prognosis factor for patients with RC who received Pre-RT in stage ypI (χ2 = 7.080; *P* = 0.008), ypII (χ2 = 20.806; *P* < 0.001) and ypIII (χ2 = 33.138; *P* < 0.001), respectively. It might provide more accurate prognostic information than the N stage alone in patients with Pre-RT.

This study has several limitations. First, the SEER database does not include information of therapeutic options such as detailed information of chemotherapy, targeted therapy, immunotherapy, recurrence and metastasis, which may also impact patients’ prognosis. Second, this data lack detailed description of preoperative clinical grading and the information about tumor and LN recession response to treatment, and our analyses could not adjust for these potential confounding factor. Third, different operative approaches, doctors and even pathologist would affect the detective rate of total LN and metastatic LN, but the SEER do not include these information [10]. Although future clinical research will be required to confirm the role of NLN, our study has its convincing power for it is one of the largest population based study until now.

## Conclusions

Our results firmly demonstrated that NLN count was an independent prognostic factor for patients with RC who received Pre-RT. It could provide more accurate prognostic information for RC patients with Pre-RT (stage ypI, ypII and ypIII, respectively).

## Abbreviations

LN: Lymph node
NLN: Negative lymph node
Pre-RT: Preoperative radiotherapy
RC: Rectal cancer
SEER: Surveillance, Epidemiology, and End Results Program
